# Cycling in older adults: a scoping review

**DOI:** 10.3389/fspor.2023.1157503

**Published:** 2023-06-29

**Authors:** Mohammadhossein Kardan, Taiyeba Akter, Mehvish Iqbal, Antonina Tcymbal, Sven Messing, Peter Gelius, Karim Abu-Omar

**Affiliations:** Department of Sport Science and Sport, Friedrich-Alexander-Universität Erlangen-Nürnberg, Erlangen, Germany

**Keywords:** cycling, older adults, physical activity, safety, active transportation

## Abstract

**Introduction:**

Regular physical activity provides many health benefits to older adults. As a well-known form of physical activity, cycling can be an appropriate means for older people to meet WHO recommendations and to improve their health. In addition, cycling can help to protect the environment and reduce greenhouse gas emissions. The primary aim of this scoping review is to identify the currently available scientific evidence and gaps of research in this field.

**Methods:**

A systematic search in seven databases resulted in 7,192 studies. After the exclusion of duplicates, studies were screened by two independent reviewers in a two-stage process. Based on previously defined inclusion criteria, 123 studies were included. Data extraction was based on a descriptive analytical method, and seven categories for the main topics of studies were developed. Data were extracted by three reviewers to analyze different characteristics of included articles such as age range, study design, data type, gender, type of bicycle, and country of origin.

**Results:**

The included studies covered the following topics: (1) traffic safety, (2) cycling as physical activity or for transport, (3) health benefits, (4) environmental factors, (5) facilitators and barriers, (6) application of technology and (7) promotion of cycling. Results show that the majority of studies were performed in both younger (60–79 years) and older (80+ years) adults. Most studies had an observational study design, used conventional bicycles, and were based on quantitative methods. Researchers from the United States, Netherlands, and Japan published the highest number of studies related to cycling.

**Discussion:**

Traffic safety was the most prevalent focus of the included studies. Gaps were identified with regard to studies focusing on the promotion of cycling, application of technology, as well as facilitators and barriers of cycling. While research on traffic safety should continue to be a high priority for public health, potentially more research should focus on how to get older people to bicycle more. This is warranted by the proven individual and planetary health benefits of cycling and the urgency of combating climate change.

## 1. Introduction

Regular physical activity (PA) provides many health benefits to older adults that affect all-cause mortality, cardiovascular diseases, hypertension, cancer, diabetes mellitus type 2, anxiety, depression, cognitive conditions, sleep, the risk of falls and falls-related injuries, bone density, and functional abilities (1). Due to these important health benefits, the World Health Organization (WHO) and national health authorities have adopted evidence-based recommendations on the volume and intensity of PA for different age groups, including older adults (1–3). Despite the well-proven health benefits of PA in older adults, study results from 122 countries show that, across WHO regions, physical inactivity in this age group is more prevalent in comparison to other age groups (4). In addition, according to a systematic review most studies show that between 20% and 60% of older adults fulfill WHO's PA recommendations (5).

As part of PA recommendations, cycling is often recommended for older adults (1, 2). Scientific evidence shows that cycling can be an appropriate training to reduce the fear of falling (6), fat mass, high blood pressure, and cholesterol in older populations (7). It also leads to fitness benefits (8) while improving the overall quality of life (9). From a functional point of view, cycling enhances skeletal muscle power and endurance, gait parameters, general functional performance (10), stepping times, one leg stance, and step response times (11). For achieving these benefits, interventions can use regular (outdoor) or stationary (indoor) bikes. In this review, we focus only on regular bikes and use the term cycling only in this context.

According to available studies, cycling has multiple benefits. For example, a bicycle-based intervention was shown to positively affect metabolic parameters, such as physical fitness level, total fat-free mass, and fasting plasma insulin levels, in rural Indian men (12). Other studies proved that increasing the level of cycling caused a reduction in the total number of road accidents (13, 14). It has also been shown that cycling can reduce the risk of falls (6, 15) and improve the mental health score in older adult populations (16). Furthermore, studies indicate that cycling in older adults can significantly improve participants' happiness, does not cause pain, and is associated with maintaining quality of life (17).

Despite these benefits, there is still potential to increase the number and proportion of older adults cycling. According to a study, 6% of overall urban transportation globally is currently carried out by bicycle, but this percentage could be increased to over 15% by 2050 (18). Another study showed specifically for older adults that 12%–24% of trips are made by bicycle in Germany, Denmark, and the Netherlands (19). In the United States, however, the overall percentage of bicycle use is comparatively low (1%), and older adults are the age group least likely to use a bicycle (20). Another study asserted that there is currently an increasing trend towards bicycle use in the United States (21).

A primary concern for older individuals has been the safety of cycling in order to avoid injuries and crashes (22), particularly as older populations have higher rates of cycling accidents (23). Some authors (24, 25) have highlighted safety and traffic as the most important concerns regarding cycling in older adults. Both cycling infrastructure (26, 27) and the built environment in general (28–33) have been shown to affect cycling behavior in older adults. Although cycling is generally highly recommended and is considered an advantageous way of transportation, most individuals choose other types of transportation. This holds true even in the Netherlands, a country with a highly developed cycling infrastructure and well-known tradition of cycling (34).

It has been suggested that psychological (35, 36), personal, and social (37) factors may facilitate or hinder cycling in older adults. To overcome such barriers, active strategies to promote cycling among older adults need to be developed. This is particularly important as cycling is not only a way to improve public health (8) but also a means to reduce pollution and greenhouse gases (38) and to support climate action (39, 40). The WHO's Global Action Plan on PA describes cycling as a “key means of transportation” that enables “regular PA on a daily basis” (1). Several interventions to promote cycling have been shown to be effective (41), and public transport policies, such as the development of infrastructures and active travel programs, can increase population PA levels (42). This research is complemented by success stories from cities that managed to increase bicycle use, for instance by integrating cycling into public transport, increasing the cost of car use, and clarifying cyclists' legal rights (43, 44).

However, there is currently a limited understanding of what research in the interdisciplinary field of cycling looks like in its entirety and how well it aligns with the potential of cycling as a means of PA promotion. Therefore, we conducted a scoping review that intends to complement existing reviews that focus on specific aspects of cycling, such as the links between the physical environment and active travel in older adults (45).

## 2. Method

This scoping review was conducted based on the framework developed by Arksey and O'Malley (46) and expanded by Levac et al. (47). The review included five key stages: (1) Identifying the research question, (2) identifying relevant studies, (3) study selection, (4) charting the data, and (5) collating, summarizing, and reporting the results. The optional sixth stage “consulting with stakeholders to inform or validate study findings” was not conducted.

### 2.1. Identifying the research question

This review aims (a) to scope the available evidence on cycling in older adults to identify its main topic as well as potential gaps in this field of research and (b) to analyze the included articles in terms of age range, study design, data type, gender, type of bicycle, and country of origin. The review focuses solely on general/outdoor cycling, as stationary indoor cycling is substantially different in terms of environmental conditions, biomechanics of pedaling, safety, feasibility, and physiological or psychological response (48–52).

### 2.2. Identifying the relevant studies

We searched for studies related to cycling in older adults published in English and German before December 22nd, 2021. The search for relevant studies was performed in seven databases: PubMed, Web of Science, Scopus, Cochrane library, SportDiscus, CINAHL, and PsychInfo. The search strategy was developed by all authors and consulted with a librarian. The search strategy included the following keywords related to cycling in older adults: (“old people” or “older people” or “elderly” or “elders” or “aging” or “ageing” or “old men” or “old women” or “older persons” or “older adults” or “seniors”) and (“bicycling” or “cycling” or “biking” or “bike” or “bicycle”). A combination of different MeSH terms and free text was used to search databases.

### 2.3. Study selection

Records were managed in the Covidence systematic review software (www.covidence.org). Duplicates were removed automatically. The screening process involved two stages based on inclusion and exclusion criteria. In the first stage, articles were screened by title and abstract, while the second stage included selection of studies by reading the full texts. The inclusion criteria were:

1. The study includes human subjects aged 60 years and/or older.
2. Cycling is one of the main components of the study or the study objective is related to cycling (general/outdoor cycling).
3. The study investigates one or more of the following:
  - the health effects of cycling,
  - injuries and/or risk of injuries caused by cycling (aspects of safety),
  - factors that determine if a person bicycles outdoors and/or the prevalence of cycling,
  - the promotion of cycling for health reasons.
4. The study was published in a peer-reviewed journal (study protocols, letters, commentaries, and conference abstracts were excluded).
5. Cycling was not used as a means to investigate a different purpose of study, e.g., to test endurance, heart rate, the training effects of cycling, or as a warm-up.

**Figure 1:**
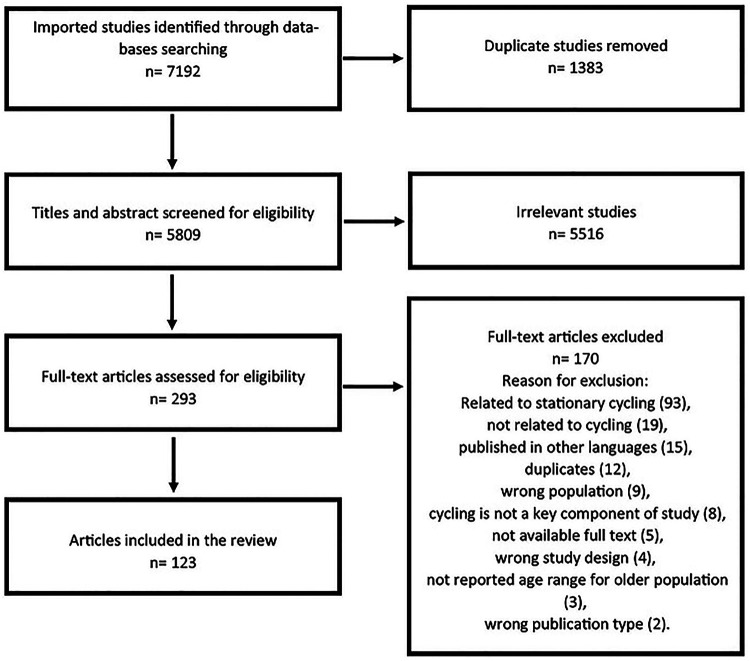
Study selection.

Figure 1 shows the stages of the search and retrieval processes of this study. The database search resulted in 7,192 studies. After removing duplicates, 5,809 studies remained. Three reviewers screened titles and abstracts, and each study was screened independently by two reviewers (MK, TA, & ME). Disagreements between the reviewers were discussed until a consensus was reached.

After title and abstract screening, the full texts of 293 references were further screened to determine their eligibility for inclusion. Each study was screened independently by two reviewers (MK & TA), and conflicts were discussed until a consensus was reached. To avoid bias, reviewers used the internet-based software Covidence, which provides random access to articles for reviewers.

### 2.4. Charting the data

A descriptive analytical method (53) was used as a basis for developing categories to structure the included studies according to their main topic. Categories were developed by a multidisciplinary group consisting of all authors (with expertise in sociology, political science, physiotherapy, medicine, and psychology) through regular meetings and discussions. Studies could be assigned to more than one category. Data extraction included the following items: title, author name, year of publication, country of origin, gender of population, study design (observational studies, interventional studies, reviews), main topic of study, type of bicycle (conventional outdoor bicycle, e-bike, pedelec), type of data gathering and analysis (qualitative, quantitative, or mix of both), and age range of the population.

## 3. Results

### 3.1. Reviews

One review was identified. In this systematic review and meta-analysis, the neighborhood physical environment and active travel in older adults were analyzed based on 42 quantitative studies. The results show that there are strong links between the neighborhood's physical environment and active travel in older adults. In particular, the review identified positive associations with total walking for residential density/urbanization, walkability, street connectivity, access to destinations/services, land use mix, pedestrian-friendly features and access to several types of destinations (45).

### 3.2. Single studies

The study characteristics of the 122 single studies are presented in Table 1. Out of these studies, 107 (87.70%) focused on all older adults (60 years old and over) and 15 (12.30%) on the lower age range of this population (60–79 years old). There were three (2.46%) studies focusing specifically on men, six (4.92%) on women, and 113 (92.62%) on both genders. 114 studies (93.44%) were based on an observational study design and eight (6.56%) were interventional (experimental). 109 studies (89.34%) were based on quantitative data, seven (5.74%) on qualitative data, and six (4.92%) mixed quantitative and qualitative data. 109 studies (89.34%) were related to only conventional outdoor bicycles, four (3.28%) to only e-bikes/pedelecs, and nine (7.38%) to both conventional outdoor bicycles and e-bikes/pedelecs.

**Table 1:** Characteristics of 122 included studies.

Characteristics of studies	Number of studies (*n*), percentage (%)
Age range	
Only younger older adults (60–79 years old)	15 (12.30%)
Only older older adults (80 years old and over)	0 (0.00%)
All older adults	107 (87.70%)
Study design	
Observational studies	114 (93.44%)
Interventional studies	8 (6.56%)
Data type	
Quantitative	109 (89.34%)
Qualitative	7 (5.74%)
Mixed method	6 (4.92%)
Gender	
Both genders	113 (92.62%)
Women only	6 (4.92%)
Men only	3 (2.46%)
Type of bicycle	
Only conventional bicycle	109 (89.34%)
Only e-bike	4 (3.28%)
Both conventional and e-bike/pedelec	9 (7.38%)

**Figure 2:**
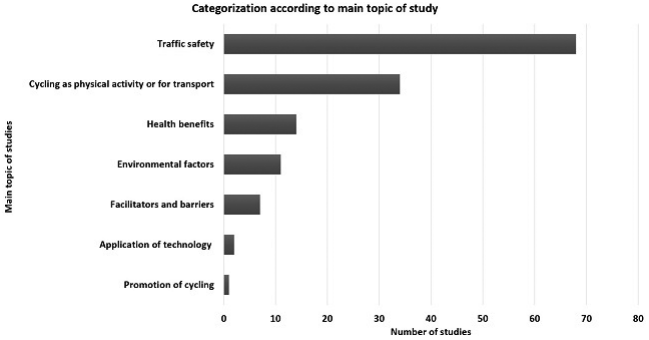
Categorization of studies on base of main topics of study.

Figure 2 shows the main topics of studies. The majority of publications were related to traffic safety, including the use of cycling helmets with 68 studies (55.74%), followed by cycling as PA and a means of transport with 34 studies (27.87%), the health benefits of cycling with 14 studies (11.48%), environmental factors with eleven studies (9.02%), facilitators and barriers of cycling with seven studies (5.74%), the application of technology with two studies (1.64%), and the promotion of cycling with one study (0.82%).

**Figure 3:**
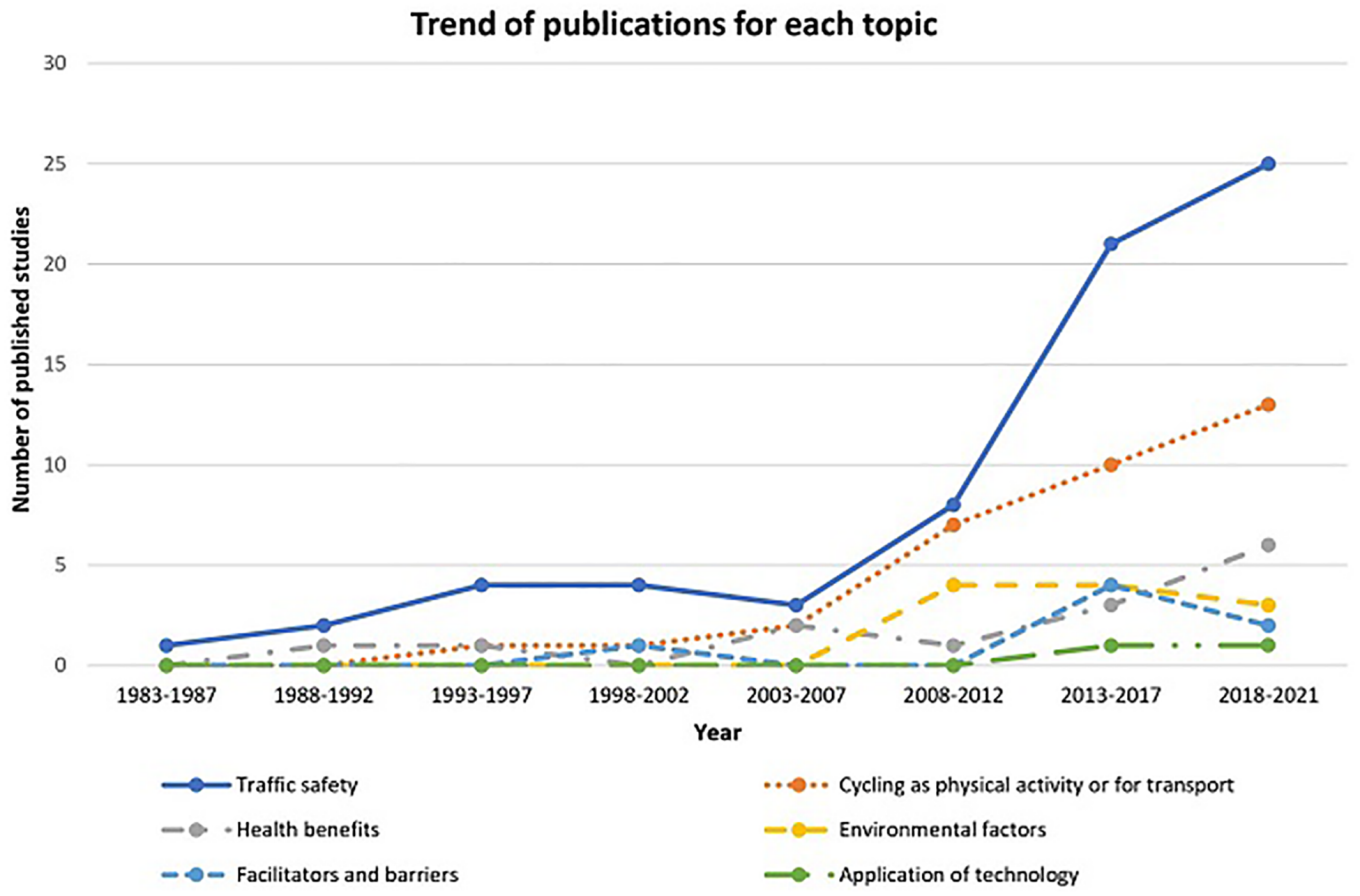
Trend on published studies during the time.

Figure 3 illustrates the growing number of publications over time. For each category, the highest number of new publications was identified for the period 2013–17 or 2018–21. Across all categories, 99 (81.15%) of the included studies were published in the last decade (2013–2021).

**Figure 4:**
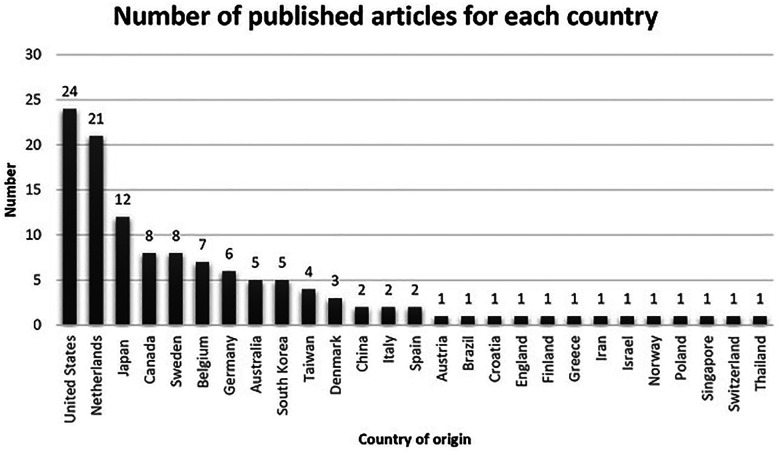
Number of published studies per country.

Figure 4 shows the number of published studies by country. The United States (24 studies), the Netherlands (21 studies), and Japan (twelve studies) had the highest number of publications, followed by Canada and Sweden (eight studies each), Belgium (seven studies), Germany (six studies), Australia and South Korea (five studies each), Taiwan (four studies), Denmark (three studies), China, Italy, and Spain (two studies each), and Austria, Brazil, Croatia, England, Finland, Greece, Iran, Israel, Norway, Poland, Singapore, Switzerland, and Thailand (one study each).

Table 2 shows the main topics of studies for the top three countries (United States, The Netherlands, Japan) in terms of quantity and contents of publications. The highest number of published studies in the United States and the Netherlands were related to traffic safety, while the highest number of studies from Japan was related to cycling as PA or for transport. The United States and the Netherlands had four and three studies on health benefits, respectively, while Japan had no study on this topic. The Netherlands had three studies related to environmental factors affecting cycling, and the United States and Japan had one study each focusing on this topic. Regarding facilitators and barriers, there was one study from the United States and no studies from the Netherlands and Japan. With regards to the application of technology, the Netherlands had two studies, while the United States and Japan had no studies on this topic. Moreover, none of the three countries had a study focusing on the promotion of cycling.

**Table 2:** Main topics of studies published in United States, The Netherlands, and Japan.

Main topic of study	United States	The Netherlands	Japan
Traffic safety	14	11	5
Cycling as physical activity or for transport	5	5	7
Health benefits of cycling	4	3	0
Environmental factors	1	3	1
Facilitators and barriers of cycling	1	0	0
Application of technology	0	2	0
Promotion of cycling	0	0	0

## 4. Discussion

### 4.1. Summary

The primary objective of this scoping review was to identify studies dealing with cycling in older adults. Overall, 123 studies were identified, most of which used observational designs, while there were few intervention studies and only one review. The studies could be categorized as pertaining to seven main topics, with more than two-thirds focusing on traffic safety. Other topics investigated more frequently include cycling as PA or for transport, health benefits, and environmental factors. By country, almost half the studies were conducted by research teams located in the United States, the Netherlands, and Japan. Time series analysis indicates that research interest in cycling among older people increased markedly after 2007.

### 4.2. Research perspectives on cycling among older adults

We observed a heightened interest in the safety aspects of cycling (24, 25, 54). By contrast, only one of the included studies dealt with the promotion of cycling among older people. This is surprising, especially when one considers that active transport (cycling and walking) has been shown to have numerous health benefits (1, 6, 7, 10, 11) and can contribute to people reaching recommended PA levels (1, 2). In addition, cycling has important environmental co-benefits (38, 40). People who engage in active transport rather than using a motorized vehicle help reduce air pollution in cities (38). Cycling in cities has also been associated with making cities safer and improving the self-reported quality of life of residents (9, 14). Likewise, from an economic point of view, a study focusing on the European Union showed each kilometer driven by car causes external costs of €0.11, while cycling represents benefits of €0.18. Also, the costs of automobility are about €500 billion per year, while the positive health effects of cycling amount to external benefits worth €24 billion per year (55).

Evidence has shown a positive association between the extent of cycling paths and non-recreational cycling (56). Environmental factors such as presence of cycling routes or paths, separation of cycling from other traffic, high population density, short trip distance, and proximity of cycling paths or green spaces are positively associated with cycling (57). These studies suggest that cycling can be promoted as a cheap way of transportation by investing in infrastructures.

Currently, prevalence rates of cycling among older people are quite low in many countries, and even in bike-friendly countries only a minority of older people report to cycle regularly (34). In France, it has been demonstrated that increasing the uptake of cycling among older people would support efforts to lower carbon emissions and adherence to the Paris Agreement (58). It can be assumed that the same might hold true for other nations. As such, the results presented here serve as a call to increase research efforts on how to promote cycling among older people. While research on safety issues should continue to be a high priority for public health, this should not deter scholars from conducting more research on how to get older people to cycle more. After all, research has shown that, the more people use bicycles, the safer cycling becomes for everybody involved (14).

### 4.3. Country differences in research on cycling among older adults

The majority of studies were conducted in high-income countries such as the United States, the Netherlands, or Japan. Among these three, the Netherlands is most prominently associated with being a “bicycling country” (59), hinting that the rate of cycling in a given country might not be able to fully explain who is engaging in research on this topic. Notably in this regard, two other countries often named as cycling-friendly countries—Denmark and Germany—have produced only a limited amount of research on this topic.

However, it seems to be highly relevant to conduct more research on cycling among older adults in low- and middle-income countries. It is a general problem of PA-related research that there is a large gap in the number of publications between high- and low-income countries (60). From a global health perspective (61, 62) this raises the question whether the available evidence is even applicable to low- and middle-income countries, which might not necessarily have an appropriate cycling infrastructure. Furthermore, geographical, political, or cultural differences might require specific research on cycling in these countries.

### 4.4. Policy context

The results indicate that research on safety issues regarding cycling is important. Also, from a political perspective, the importance of road safety in the context of PA promotion is reflected in documents such as WHO's Global Action Plan on Physical Activity (WHO 2018) and its European Physical Activity Strategy (WHO 2016). These documents call for the implementation of policy actions to improve the safety of cyclists (WHO 2018) and for identifying linkages between PA promotion and road safety strategies (WHO 2016). In addition, policy documents highlight the importance of a cycling network infrastructure (WHO 2016, 2018), pre- and in-service training of professionals in the transport sector on PA promotion (WHO 2018), and the removal of barriers for disadvantaged groups (WHO 2016). An in-depth analysis of the identified studies on facilitators and barriers of cycling for older adults could help to identify target group-specific barriers for cycling and develop appropriate strategies to remove them.

### 4.5. Limitations

This study was limited to published studies in English or German, and including studies written in other languages might have changed the nature of the results. Also, we acknowledge that the categorization of the identified studies by research topic was not always easy, since some studies covered more than just one topic (e.g., environmental factors and facilitators/barriers). Furthermore, this review focused exclusively on scientific studies and did not include other types of publications, such as policies and intervention reports, that may also serve to promote cycling among older adults.

### 4.6. Conclusion

To our knowledge, this is the first scoping review in the field of cycling in older adults which covers a broad range of articles in order to identify the state of the evidence on cycling in older adults. Traffic safety was the most prevalent topic of the included studies. The number of studies being published has increased over time, but the rate of increase for each main study topic was different. For some topics, such as the promotion of cycling and health benefits, only a few published studies were identified despite their well-documented importance. The majority of studies were conducted in a small number of countries. Studies from additional countries and world regions may be required to account for different geographical, infrastructural and cultural contexts. Moreover, for some study topics, such as environmental factors, facilitators and barriers, application of technology, and promotion of cycling, only a few published studies were identified, warranting further attention to these topics in future research. The growing tendency towards ownership and use of e-bikes, pedelecs, and recumbent bicycles, also seems to call for conducting more research in these fields. In addition, given the very low numbers of qualitative and review study designs identified in this paper, further research using non-quantitative designs may enrich the evidence on how to promote cycling. Finally, most studies had observational study designs, while only a few interventional studies were conducted. The urgent need to promote cycling requires more interventional studies to produce evidence on how to provide ideal conditions for cycling and maximize the desired effects.
