# Supplementary material for: Cycling in older adults: a scoping review
Source: Front Sports Act Living. 2023 Jun 29;5:1157503. doi: 10.3389/fspor.2023.1157503 (PMC10338922; doi:10.3389/fspor.2023.1157503)
Supplement: Supplementary file 1 [file Table1.docx]

**Supplementary material 1: Data extraction for all 122 included single studies**

| **No** | **Author (Year)** | **Country of origin/author affiliation** | **Gender** | **Study design** | **Main topic of study** | **Type  of  Bike** | **Data type** | **Age range** |
| --- | --- | --- | --- | --- | --- | --- | --- | --- |
| 1 | Abe et al. (2018) | Japan | Both | Obs | Cycling as PA or for transport | CB | Qt | All older adults |
| 2 | Allan et al. (2013) | United States | Both | Obs | Traffic safety | CB | Qt | All older adults |
| 3 | Aslan et al. (2020) | United States | Both | Obs | Health benefits | CB | Qt | All older adults |
| 4 | Babatsikou et al. (2012) | Greece | Both | Obs | Cycling as PA or for transport | CB | Qt | All older adults |
| 5 | Batcir and Melzer (2018) | Israel | Both | Obs | Health benefits, traffic safety | CB | Qt | All older adults |
| 6 | Bertera (2003) | United States | Both | Obs | Health benefits | CB | Qt | All older adults |
| 7 | Bijnen et al. (1996) | Netherlands | Men only | Obs | Cycling as PA or for transport,  health benefits | CB | Qt | All older adults |
| 8 | Boele-Vos et al. (2017) | Netherlands | Both | Obs | Traffic safety | CB and P | Qt | All older adults |
| 9 | Boufous et al. (2021) | Australia | Both | Obs | Facilitators and barriers | CB | Qt | All older adults |
| 10 | Buehler and Pucher (2017) | United States | Both | Obs | Traffic safety | CB | Qt | All older adults |
| 11 | Bungum and Bungum (2003) | United States | Both | Obs | Traffic safety | CB | Qt | All older adults |
| 12 | Caruso et al. (2015) | Italy | Women only | Obs | Traffic safety | CB | Qt | Only younger older adults (60-79 years) |
| 13 | Chad et al. (2005) | Canada | Both | Obs | Cycling as PA or for transport | CB | Qt | All older adults |
| 14 | Champahom et al. (2020) | Thailand | Both | Obs | Cycling as PA or for transport | CB | Qt | All older adults |
| 15 | Chang et al. (2020) | Taiwan | Both | Obs | Traffic safety | CB | Qt | All older adults |
| 16 | Chen et al. (2015) | Taiwan | Both | Obs | Cycling as PA or for transport | CB | Qt | All older adults |
| 17 | Chen et al. (2013) | United States | Both | Obs | Traffic safety | CB | Qt | All older adults |
| 18 | Choi et al. (2021) | South Korea | Both | Obs | Traffic safety | CB | Qt | All older adults |
| 19 | Chong et al. (2010) | Australia | Both | Obs | Traffic safety | CB | Qt | All older adults |
| 20 | Cousins (2000) | Canada | Women only | Obs | Facilitators and barriers | CB | Ql | All older adults |
| 21 | Das et al. (2019) | United States | Both | Obs | Traffic safety | CB | Qt | All older adults |
| 22 | DiMaggio et al. (2020) | United States | Both | Obs | Traffic safety | E | Qt | All older adults |
| 23 | Dubbeldam, Baten, Buurke, et al. (2017) | Netherlands | Both | Int | Traffic safety, application of technology | CB | Qt | All older adults |
| 24 | Dubbeldam, Baten, Straathof, et al. (2017) | Netherlands | Both | Obs | Traffic safety | CB | Qt | All older adults |
| 25 | Duda (2008) | Poland | Both | Obs | Cycling as PA or for transport | CB | Qt | Only younger older adults (60-79 years) |
| 26 | Dupont et al. (1996) | Denmark | Both | Obs | Traffic safety | CB | Qt | All older adults |
| 27 | Edwards and Mason (2014) | United States | Both | Obs | Traffic safety, Health benefits | CB | Qt | All older adults |
| 28 | Ekman et al. (1997) | Sweden | Both | Obs | Traffic safety | CB | Qt | All older adults |
| 29 | Ekman et al. (2001) | Sweden | Both | Obs | Traffic safety | CB | Qt | All older adults |
| 30 | Eluru et al. (2008) | United States | Both | Obs | Traffic safety | CB | Qt | All older adults |
| 31 | Engbers et al. (2018) | Netherlands | Both | Obs | Traffic safety | CB | Qt | All older adults |
| 32 | Fishman et al. (2015) | Netherlands | Both | Obs | Cycling as PA or for transport | CB | Qt | All older adults |
| 33 | Frith and Loprinzi (2018) | United States | Both | Obs | Health benefits | CB | Qt | All older adults |
| 34 | Garcia et al. (2013) | Brazil | Both | Obs | Traffic safety | CB | Qt | All older adults |
| 35 | Grimes et al. (2020) | United States | Both | Obs | Facilitators and barriers | CB | Qt | All older adults |
| 36 | Handa and Mitobe (2020) | Japan | Both | Int | Traffic safety | CB | Qt | Only younger older adults (60-79 years) |
| 37 | Harvey et al. (2018) | Australia | Both | Obs | Health benefits, traffic safety | CB | Qt | All older adults |
| 38 | Hertach et al. (2018) | Switzerland | Both | Obs | Traffic safety |  | Qt | All older adults |
| 39 | Hinrichs et al. (2010) | Germany | Both | Obs | Cycling as PA or for transport | CB | Qt | All older adults |
| 40 | Hosseinpour et al. (2021) | United States | Both | Obs | Traffic safety | CB | Qt | All older adults |
| 41 | Hu and Baker (2010) | China | Both | Obs | Traffic safety | CB | Qt | All older adults |
| 42 | Jochems et al. (2021) | Netherlands | Both | Obs | Traffic safety | CB | Qt | All older adults |
| 43 | Johansson et al. (2019) | Denmark | Both | Obs | Cycling as PA or for transport | CB | Qt | All older adults |
| 44 | Jung et al. (2018) | Japan | Both | Obs | Cycling as PA or for transport | CB | Qt | Only younger older adults (60-79 years) |
| 45 | Kaimila et al. (2013) | Japan | Both | Obs | Traffic safety | CB | Qt | Only younger older adults (60-79 years) |
| 46 | Kammerlander et al. (2012) | Austria | Both | Obs | Traffic safety | CB | Qt | All older adults |
| 47 | Kaplan et al. (2014) | Denmark | Both | Obs | Traffic safety | CB | Qt | All older adults |
| 48 | Kay et al. (2019) | United States | Both | Obs | Traffic safety | CB | Qt | All older adults |
| 49 | Kemperman and Timmermans (2009) | Netherlands | Both | Obs | Environmental factors | CB | Qt | All older adults |
| 50 | Kim et al. (2015) | South Korea | Women only | Obs | Health benefits | CB | Qt | All older adults |
| 51 | Kim et al. (2019) | South Korea | Both | Obs | Traffic safety | CB | Qt | All older adults |
| 52 | Kim et al. (2017) | South Korea | Both | Obs | Traffic safety | CB | Qt | All older adults |
| 53 | Kingma (1994) | Netherlands | Both | Obs | Traffic safety | CB | Qt | All older adults |
| 54 | Koolhaas et al. (2016) | Netherlands | Both | Obs | Health benefits | CB | Qt | All older adults |
| 55 | Kramer et al. (2013) | Netherlands | Both | Obs | Cycling as PA or for transport | CB | Qt | All older adults |
| 56 | Kremers et al. (2012) | Netherlands | Both | Obs | Traffic safety, health benefits | CB | Qt | Only younger older adults (60-79 years) |
| 57 | Lustenberger et al. (2010) | United States | Both | Obs | Traffic safety | CB | Qt | All older adults |
| 58 | Lutz and Kreidel (1988) | Germany | Both | Obs | Traffic safety | CB | Qt | All older adults |
| 59 | McNiel and Westphal (2020) | United States | Both | Obs | Cycling as PA or for transport | CB | Ql | All older adults |
| 60 | Mertens et al. (2019) | Belgium | Both | Obs | Cycling as PA or for transport | CB | Qt | All older adults |
| 61 | Meusel and Rieder (1999) | United States | Both | Obs | Traffic safety | CB | Qt | All older adults |
| 62 | Missoni and Kern (2003) | Croatia | Both | Obs | Traffic safety | CB | Qt | All older adults |
| 63 | Mittleman et al. (1989) | Canada | Both | Obs | Health benefits | CB | Qt | Only younger older adults (60-79 years) |
| 64 | Næss et al. (2020) | Norway | Both | Obs | Traffic safety | CB | Qt | All older adults |
| 65 | Noordzij et al. (2021) | Netherlands | Both | Obs | Cycling as PA or for transport | CB | Qt | Only younger older adults (60-79 years) |
| 66 | Pai et al. (2017) | Taiwan | Both | Obs | Traffic safety | CB | Qt | All older adults |
| 67 | Pai et al. (2018) | Taiwan | Both | Obs | Traffic safety | CB | Qt | All older adults |
| 68 | Petzoldt et al. (2017) | Germany | Both | Obs | Traffic safety | E and CB | Qt | All older adults |
| 69 | Prins and Van Lenthe (2015) | Netherlands | Both | Obs | Environmental factors | CB | Qt | All older adults |
| 70 | Pritchard et al. (2014) | Australia | Both | Obs | Cycling as PA or for transport | CB | Qt | All older adults |
| 71 | Pucher et al. (2011) | United States | Both | Obs | Cycling as PA or for transport | CB | Qt | All older adults |
| 72 | Pulakka et al. (2020) | Finland | Both | Obs | Cycling as PA or for transport | CB | Qt | Only younger older adults (60-79 years) |
| 73 | Reuvers et al. (2020) | Netherlands | Both | Obs | Traffic safety | CB | Qt | All older adults |
| 74 | Richter et al. (2005) | Germany | Both | Obs | Traffic safety | CB | Qt | All older adults |
| 75 | Rodgers (1997) | United States | Both | Obs | Traffic safety | CB | Qt | All older adults |
| 76 | Rolfsman et al. (2012) | Sweden | Both | Obs | Traffic safety | CB | Qt | All older adults |
| 77 | Rosenkvist et al. (2014) | Sweden | Both | Obs | Environmental factors | CB | Qt  and Ql | All older adults |
| 78 | Ryan et al. (2016) | Sweden | Both | Obs | Cycling as PA or for transport | CB | Qt  and Ql | All older adults |
| 79 | Sadeghvaziri and Tawfik (2020) | United States | Both | Obs | Cycling as PA or for transport | CB | Qt | All older adults |
| 80 | Sakurai et al. (2019) | Japan | Women only | Obs | Traffic safety | CB | Qt | All older adults |
| 81 | Sakurai et al. (2016) | Japan | Women only | Obs | Cycling as PA or for transport, traffic safety | CB | Qt | All older adults |
| 82 | Scheiman et al. (2010) | Sweden | Both | Obs | Traffic safety | CB | Qt | All older adults |
| 83 | Shaer and Haghshenas (2021) | Iran | Both | Obs | Cycling as PA or for transport | CB | Qt | All older adults |
| 84 | Sjögren and Björnstig (1991) | Sweden | Both | Obs | Traffic safety | CB | Qt | All older adults |
| 85 | Son et al. (2018) | South Korea | Both | Obs | Traffic safety | CB | Qt | All older adults |
| 86 | Stones and Hartin (2017) | Canada | Both | Obs | Facilitators and barriers | CB | Qt | All older adults |
| 87 | Takahashi et al. (2012) | United States | Both | Obs | Environmental factors | CB | Qt | All older adults |
| 88 | Tanabe (2019) | Japan | Both | Obs | Traffic safety | CB | Qt | All older adults |
| 89 | Tredray (2019) | Canada | Both | Int | Promotion of cycling | CB and E | Ql | All older adults |
| 90 | Tsuji et al. (2015) | Japan | Men only | Obs | Traffic safety | CB | Ql | Only younger older adults (60-79 years) |
| 91 | Tsunoda et al. (2015) | Japan | Both | Obs | Cycling as PA or for transport | CB | Qt | All older adults |
| 92 | Tsunoda et al. (2021) | Japan | Both | Obs | Cycling as PA or for transport | CB | Qt | All older adults |
| 93 | Tsunoda et al. (2012) | Japan | Both | Obs | Cycling as PA or for transport, environmental factors | CB | Qt | All older adults |
| 94 | Twisk et al. (2017) | Netherlands | Both | Int | Traffic safety | CB and P | Qt | All older adults |
| 95 | Ulak et al. (2018) | United States | Both | Obs | Traffic safety | CB | Qt | All older adults |
| 96 | Useche et al. (2018) | Spain | Both | Obs | Traffic safety | CB | Qt | Only younger older adults (60-79 years) |
| 97 | Valent et al. (2002) | Italy | Both | Obs | Traffic safety | CB | Qt | All older adults |
| 98 | Van Cauwenberg, Clarys, et al. (2018) | Belgium | Both | Obs | Environmental factors | CB | Ql | All older adults |
| 99 | Van Cauwenberg, De Bourdeaudhuij, et al. (2019a) | Belgium | Both | Obs | Traffic safety | E | Qt | All older adults |
| 100 | Van Cauwenberg et al. (2012) | Belgium | Both | Obs | Environmental factors | CB | Qt | All older adults |
| 101 | Van Cauwenberg, De Bourdeaudhuij, et al. (2019b) | Belgium | Both | Obs | Environmental factors | CB | Qt | All older adults |
| 102 | Van Cauwenberg, De Bourdeaudhuij, et al. (2018) | Belgium | Both | Obs | Cycling as PA or for transport, health benefits | E | Qt | All older adults |
| 103 | Van Cauwenberg, Schepers, et al. (2019) | Belgium | Both | Obs | Cycling as PA or for transport,  health benefits | E and CB | Qt | All older adults |
| 104 | Vance et al. (2008) | England | Both | Obs | Cycling as PA or for transport | CB | Qt | All older adults |
| 105 | Van den Berg et al. (2011) | Netherlands | Both | Obs | Cycling as PA or for transport | CB | Qt | All older adults |
| 106 | Värnild et al. (2020) | Sweden | Both | Obs | Traffic safety | CB | Qt | All older adults |
| 107 | Velasco et al. (2015) | Spain | Both | Obs | Traffic safety, Facilitators and barriers | CB | Qt | All older adults |
| 108 | Vlakveld et al. (2015) | Netherlands | Both | Int | Traffic safety | CB and E | Qt | Only younger older adults (60-79 years) |
| 109 | Voss et al. (2016) | Canada | Both | Obs | Cycling as PA or for transport | CB | Qt | All older adults |
| 110 | Walsh et al. (2001) | United States | Women only | Obs | Cycling as PA or for transport | CB | Qt | All older adults |
| 111 | Weijermars et al. (2016) | Netherlands | Both | Obs | Traffic safety | CB | Qt | All older adults |
| 112 | Weinz and Schönle (2001) | Germany | Both | Obs | Traffic safety | CB | Qt | All older adults |
| 113 | Weiss et al. (2018) | Germany | Both | Obs | Traffic safety | CB and E | Qt | All older adults |
| 114 | Westerhuis et al. (2021) | Netherlands | Both | Int | Application of technology, Traffic safety | CB | Qt and Ql | All older adults |
| 115 | Westerhuis et al. (2020) | Netherlands | Both | Int | Environmental factors | E and CB | Qt and Ql | All older adults |
| 116 | Whitaker (2005) | United States | Men only | Obs | Cycling as PA or for transport, health benefits | CB | Qt and Ql | Only younger older adults (60-79 years) |
| 117 | Winters, Voss, et al. (2015) | Canada | Both | Obs | Cycling as PA or for transport, environmental factors | CB | Qt | All older adults |
| 118 | Winters, Sims-Gould, et al. (2015) | Canada | Both | Obs | Facilitators and barriers, traffic safety | CB | Qt and Ql | All older adults |
| 119 | Wong (1987) | Singapore | Both | Obs | Traffic safety | CB | Qt | All older adults |
| 120 | Yamada et al. (2013) | Japan | Both | Obs | Cycling as PA or for transport | CB | Qt | All older adults |
| 121 | Zander (2013) | Australia | Both | Obs | Facilitators and barriers | CB | Ql | All older adults |
| 122 | Zhang (2016) | China | Both | Obs | Environmental factors | CB and E | Qt | Only younger older adults (60-79 years) |

Note: CB (Conventional Bicycle), E (E-bike), Int (Interventional), Obs (Observational), P (Pedelec), Ql (Qualitative), Qt (Quantitative).

**Supplementary material 2: List of included single studies**

1. Abe T, Seol J, Kim M, Okura T. The Relationship of Car Driving and Bicycle Riding on Physical Activity and Social Participation in Japanese Rural Areas. *Journal of Transport & Health* (2018) 10:315-21.

2. Allan BJ, Davis JS, Pandya RK, Jouria J, Habib F, Namias N, et al. Exploring Trauma Recidivism in an Elderly Cohort. *Journal of surgical research* (2013) 184(1):582-5.

3. Aslan DH, Collette JM, Ortega JD. Bicycling Exercise Helps Maintain a Youthful Metabolic Cost of Walking in Older Adults. *Journal of Aging and Physical Activity* (2020) 29(1):36-42.

4. Babatsikou F, Gerogianni G, Zyga S, Koutis C. Physical Activity in a Sample of Elderly Greek People: A Research Study. *Health Science Journal* (2012) 6(3):518.

5. Batcir S, Melzer I. Daily Bicycling in Older Adults May Be Effective to Reduce Fall Risks—a Case-Control Study. *Journal of aging and physical activity* (2018) 26(4):570-6.

6. Bertera EM. Physical Activity and Social Network Contacts in Community Dwelling Older Adults. *Activities, Adaptation & Aging* (2003) 27(3-4):113-27.

7. Bijnen FC, Feskens EJ, Caspersen CJ, Giampaoli S, Nissinen AM, Menotti A, et al. Physical Activity and Cardiovascular Risk Factors among Elderly Men in Finland, Italy, and the Netherlands. *American Journal of Epidemiology* (1996) 143(6):553-61.

8. Boele-Vos M, Commandeur J, Twisk D. Effect of Physical Effort on Mental Workload of Cyclists in Real Traffic in Relation to Age and Use of Pedelecs. *Accident Analysis & Prevention* (2017) 105:84-94.

9. Boufous S, Beck B, Macniven R, Pettit C, Ivers R. Facilitators and Barriers to Cycling in Older Residents of New South Wales, Australia. *Journal of Transport & Health* (2021) 21:101056.

10. Buehler R, Pucher J. Trends in Walking and Cycling Safety: Recent Evidence from High-Income Countries, with a Focus on the United States and Germany. *American journal of public health* (2017) 107(2):281-7.

11. Bungum TJ, Bungum NW. Predictors of Bicycle Helmet Usage among Seniors. *Journal of community health* (2003) 28(3):221-8.

12. Caruso G, Vitali A, del Prete F. Multiple Ruptures of the Extensor Tendons after Volar Fixation for Distal Radius Fracture: A Case Report. *Injury* (2015) 46:S23-S7.

13. Chad KE, Reeder BA, Harrison EL, Ashworth NL, Sheppard SM, Schultz SL, et al. Profile of Physical Activity Levels in Community-Dwelling Older Adults. *Medicine and science in sports and exercise* (2005) 37(10):1774.

14. Champahom T, Jomnonkwao S, Nambulee W, Klungboonkrong P, Karoonsoontawong A, Ratanavaraha V. Analyzing Transport Mode Choice for Aging Society in Thailand. *Engineering and Applied Science Research* (2020) 47(4):383-92.

15. Chang Y-H, Li C-Y, Lu T-H, Artanti KD, Hou W-H. Risk of Injury and Mortality among Driver Victims Involved in Single-Vehicle Crashes in Taiwan: Comparisons between Vehicle Types. *International journal of environmental research and public health* (2020) 17(13):4687.

16. Chen W-H, Lin W-I, Chang S-H, Mak L-C. Exploring Relationships between Physiological and Psychological Condition of Seniors and Their Mobility and Social Activity. *Transportation research record* (2015) 2537(1):103-10.

17. Chen WS, Dunn RY, Chen AJ, Linakis JG. Epidemiology of Nonfatal Bicycle Injuries Presenting to United States Emergency Departments, 2001–2008. *Academic emergency medicine* (2013) 20(6):570-5.

18. Choi Y, Lee DH, Lee JI. Patterns and Clinical Outcomes of Injuries Related to Two-Wheeled Vehicles (Bicycle and Motorcycle) in the Geriatric Population: A Nationwide Analysis in South Korea (2016–2018). *BMC geriatrics* (2021) 21(1):1-13.

19. Chong S, Poulos R, Olivier J, Watson WL, Grzebieta R. Relative Injury Severity among Vulnerable Non-Motorised Road Users: Comparative Analysis of Injury Arising from Bicycle–Motor Vehicle and Bicycle–Pedestrian Collisions. *Accident Analysis & Prevention* (2010) 42(1):290-6.

20. Cousins SOB. " My Heart Couldn't Take It" Older Women's Beliefs About Exercise Benefits and Risks. *The Journals of Gerontology Series B: Psychological Sciences and Social Sciences* (2000) 55(5):P283-P94.

21. Das S, Jha K, Fitzpatrick K, Brewer M, Shimu TH. Pattern Identification from Older Bicyclist Fatal Crashes. *Transportation research record* (2019) 2673(6):638-49.

22. DiMaggio CJ, Bukur M, Wall SP, Frangos SG, Wen AY. Injuries Associated with Electric-Powered Bikes and Scooters: Analysis of Us Consumer Product Data. *Injury prevention* (2020) 26(6):524-8.

23. Dubbeldam R, Baten C, Buurke J, Rietman J. Sofie, a Bicycle That Supports Older Cyclists? *Accident analysis & prevention* (2017) 105:117-23.

24. Dubbeldam R, Baten C, Straathof P, Buurke J, Rietman J. The Different Ways to Get on and Off a Bicycle for Young and Old. *Safety science* (2017) 92:318-29.

25. Duda B. Physical Activity and Fitness of Adults Aged 60-69 Years. *Med Sport* (2008) 24(6):379-84.

26. Dupont G, Jensen B, Hansen T. Fatal Road Traffic Accidents in People Aged 70 Years or More. *Journal of traffic medicine* (1996) 24(3-4):91-3.

27. Edwards RD, Mason CN. Spinning the Wheels and Rolling the Dice: Life-Cycle Risks and Benefits of Bicycle Commuting in the Us. *Preventive medicine* (2014) 64:8-13.

28. Ekman R, Schelp L, Welander G, Svanström L. Can a Combination of Local, Regional and National Information Substantially Increase Bicycle-Helmet Wearing and Reduce Injuries? Experiences from Sweden. *Accident Analysis & Prevention* (1997) 29(3):321-8.

29. Ekman R, Welander G, Svanström L, Schelp L, Santesson P. Bicycle-Related Injuries among the Elderly—a New Epidemic? *Public Health* (2001) 115(1):38-43.

30. Eluru N, Bhat CR, Hensher DA. A Mixed Generalized Ordered Response Model for Examining Pedestrian and Bicyclist Injury Severity Level in Traffic Crashes. *Accident Analysis & Prevention* (2008) 40(3):1033-54.

31. Engbers C, Dubbeldam R, Brusse-Keizer M, Buurke J, De Waard D, Rietman J. Characteristics of Older Cyclists (65+) and Factors Associated with Self-Reported Cycling Accidents in the Netherlands. *Transportation research part F: traffic psychology and behaviour* (2018) 56:522-30.

32. Fishman E, Böcker L, Helbich M. Adult Active Transport in the Netherlands: An Analysis of Its Contribution to Physical Activity Requirements. *PloS one* (2015) 10(4):e0121871.

33. FRİTH E, LOPRINZI PD. Individual-Specific Physical Activities on Cognitive Function among Older Adults. *Journal of Cognitive-Behavioral Psychotherapy and Research* (2018) 7(3):107-.

34. Garcia LP, Freitas LRSd, Duarte EC. Deaths of Bicycle Riders in Brazil: Characteristics and Trends During the Period of 2000-2010. *Revista Brasileira de Epidemiologia* (2013) 16:918-29.

35. Grimes A, Chrisman M, Lightner J. Barriers and Motivators of Bicycling by Gender among Older Adult Bicyclists in the Midwest. *Health Education & Behavior* (2020) 47(1):67-77.

36. Handa N, Mitobe K. Investigation of Collision Position and Orientation of Elderly Cyclist for Car‐Bicycle Collision Simulation: Factors Causing Accidents on the Side of a Cyclist When Turning Right on Two Lane Straight Road. *IEEJ Transactions on Electrical and Electronic Engineering* (2020) 15(11):1705-7.

37. Harvey S, Rissel C, Pijnappels M. Associations between Bicycling and Reduced Fall-Related Physical Performance in Older Adults. *Journal of aging and physical activity* (2018) 26(3):514-9.

38. Hertach P, Uhr A, Niemann S, Cavegn M. Characteristics of Single-Vehicle Crashes with E-Bikes in Switzerland. *Accident Analysis & Prevention* (2018) 117:232-8.

39. Hinrichs T, Trampisch U, Burghaus I, Endres HG, Klaaßen-Mielke R, Moschny A, et al. Correlates of Sport Participation among Community-Dwelling Elderly People in Germany: A Cross-Sectional Study. *European Review of Aging and Physical Activity* (2010) 7(2):105-15.

40. Hosseinpour M, Madsen TKO, Olesen AV, Lahrmann H. An in-Depth Analysis of Self-Reported Cycling Injuries in Single and Multiparty Bicycle Crashes in Denmark. *Journal of safety research* (2021) 77:114-24.

41. Hu G, Baker SP. Recent Increases in Fatal and Non-Fatal Injury among People Aged 65 Years and over in the USA. *Injury Prevention* (2010) 16(1):26-30.

42. Jochems D, van Rein E, Niemeijer M, van Heijl M, van Es MA, Nijboer T, et al. Incidence, Causes and Consequences of Moderate and Severe Traumatic Brain Injury as Determined by Abbreviated Injury Score in the Netherlands. *Scientific reports* (2021) 11(1):1-8.

43. Johansson MS, Korshøj M, Schnohr P, Marott JL, Prescott EIB, Søgaard K, et al. Time Spent Cycling, Walking, Running, Standing and Sedentary: A Cross-Sectional Analysis of Accelerometer-Data from 1670 Adults in the Copenhagen City Heart Study. *BMC public health* (2019) 19(1):1-13.

44. Jung S, Okubo Y, Osuka Y, Seino S, Park J, Nho H, et al. Older Korean Adults Have Lower Physical Function Despite Longer Exercise Times Compared to Their Japanese Counterparts: A Japan‐Korea Comparative Study. *Geriatrics & Gerontology International* (2018) 18(4):576-83.

45. Kaimila B, Yamashina H, Arai A, Tamashiro H. Road Traffic Crashes and Fatalities in Japan 2000–2010 with Special Reference to the Elderly Road User. *Traffic injury prevention* (2013) 14(8):777-81.

46. Kammerlander C, Braito M, Kates S, Jeske C, Roth T, Blauth M, et al. The Epidemiology of Sports-Related Injuries in Older Adults: A Central European Epidemiologic Study. *Aging clinical and experimental research* (2012) 24(5):448-54.

47. Kaplan S, Vavatsoulas K, Prato CG. Aggravating and Mitigating Factors Associated with Cyclist Injury Severity in Denmark. *Journal of safety research* (2014) 50:75-82.

48. Kay AB, Wilson EL, White TW, Morris DS, Majercik S. Age Is Just a Number: A Look at “Elderly” Sport-Related Traumatic Injuries at a Level I Trauma Center. *The American Journal of Surgery* (2019) 217(6):1121-5.

49. Kemperman A, Timmermans H. Influences of Built Environment on Walking and Cycling by Latent Segments of Aging Population. *Transportation research record* (2009) 2134(1):1-9.

50. Kim H-k, Kim H-S, Park M-H, Kang H-S. A Comparative Study on Cycling in Relation to the Physical Balance, Muscle Strength, Self-Esteem and Depression of Elderly Women. *Indian journal of science and technology* (2015) 8:169.

51. Kim T, Jung KY, Kim K, Yoon H, Hwang SY, Shin TG, et al. Protective Effects of Helmets on Bicycle-Related Injuries in Elderly Individuals. *Injury prevention* (2019) 25(5):407-13.

52. Kim Y-J, Seo D-W, Lee J-H, Lee Y-S, Oh B-J, Lim K-S, et al. Trends in the Incidence and Outcomes of Bicycle-Related Injury in the Emergency Department: A Nationwide Population-Based Study in South Korea, 2012-2014. *PLoS one* (2017) 12(7):e0181362.

53. Kingma J. The Aetiology of Bicycle Accidents. *Perceptual and motor skills* (1994) 79(3):1193-4.

54. Koolhaas CM, Dhana K, Golubic R, Schoufour JD, Hofman A, van Rooij FJ, et al. Physical Activity Types and Coronary Heart Disease Risk in Middle-Aged and Elderly Persons: The Rotterdam Study. *American journal of epidemiology* (2016) 183(8):729-38.

55. Kramer D, Maas J, Wingen M, Kunst AE. Neighbourhood Safety and Leisure-Time Physical Activity among Dutch Adults: A Multilevel Perspective. *International journal of behavioral nutrition and physical activity* (2013) 10(1):1-10.

56. Kremers SP, de Bruijn G-J, Visscher TL, Deeg DJ, Thomese G, Visser M, et al. Associations between Safety from Crime, Cycling, and Obesity in a Dutch Elderly Population: Results from the Longitudinal Aging Study Amsterdam. *Journal of environmental and public health* (2012) 2012.

57. Lustenberger T, Inaba K, Talving P, Barmparas G, Schnüriger B, Green D, et al. Bicyclists Injured by Automobiles: Relationship of Age to Injury Type and Severity—a National Trauma Databank Analysis. *Journal of Trauma and Acute Care Surgery* (2010) 69(5):1120-5.

58. Lutz F, St Kreidel H. Fatal Bicycle Accidents--Causes and Legal Responsibility. *Zeitschrift fur Rechtsmedizin Journal of Legal Medicine* (1988) 101(1):1-8.

59. McNiel P, Westphal J. Cycling without Age Program: The Impact for Residents in Long-Term Care. *Western journal of nursing research* (2020) 42(9):728-35.

60. Mertens L, Van Dyck D, Deforche B, De Bourdeaudhuij I, Brondeel R, Van Cauwenberg J. Individual, Social, and Physical Environmental Factors Related to Changes in Walking and Cycling for Transport among Older Adults: A Longitudinal Study. *Health & Place* (2019) 55:120-7.

61. Meusel H, Rieder H. Sport Für Ältere Bewegung—Sportarten—Training. *German Journal of Exercise and Sport Research* (1999) 29(3):364-6.

62. Missoni E, Kern J. Fatality Risk Factors for Bicyclists in Croatia. *Croatian medical journal* (2003) 44(5):610-3.

63. Mittleman K, Crawford S, Holliday S, Gutman G, Bhakthan G. The Older Cyclist: Anthropometric, Physiological, and Psychosocial Changes Observed During a Trans-Canada Cycle Tour. *Canadian Journal on Aging/La Revue canadienne du vieillissement* (1989) 8(2):144-56.

64. Næss I, Galteland P, Skaga NO, Eken T, Helseth E, Ramm-Pettersen J. The Number of Patients Hospitalized with Bicycle Injuries Is Increasing-a Cry for Better Road Safety. *Accident Analysis & Prevention* (2020) 148:105836.

65. Noordzij JM, Beenackers MA, Groeniger JO, Timmermans E, Chaix B, Doiron D, et al. Green Spaces, Subjective Health and Depressed Affect in Middle-Aged and Older Adults: A Cross-Country Comparison of Four European Cohorts. *J Epidemiol Community Health* (2021) 75(5):470-6.

66. Pai C-W, Chen Y-C, Lin H-Y, Chen P-L. A Population-Based Case-Control Study of Hospitalisation Due to Head Injuries among Bicyclists and Motorcyclists in Taiwan. *BMJ open* (2017) 7(11):e018574.

67. Pai C-W, Lin H-Y, Tsai S-H, Chen P-L. Comparison of Traffic-Injury Related Hospitalisation between Bicyclists and Motorcyclists in Taiwan. *PloS one* (2018) 13(1):e0191221.

68. Petzoldt T, Schleinitz K, Heilmann S, Gehlert T. Traffic Conflicts and Their Contextual Factors When Riding Conventional Vs. Electric Bicycles. *Transportation research part F: traffic psychology and behaviour* (2017) 46:477-90.

69. Prins RG, Van Lenthe F. The Hour-to-Hour Influence of Weather Conditions on Walking and Cycling among Dutch Older Adults. *Age and ageing* (2015) 44(5):886-90. doi: https://doi.org/10.1093/ageing/afv103.

70. Pritchard EK, Brown GT, Barker AL, Haines TP. Exploring the Association between Volition and Participation in Daily Life Activities with Older Adults Living in the Community. *Clinical Rehabilitation* (2014) 28(11):1136-44.

71. Pucher J, Buehler R, Merom D, Bauman A. Walking and Cycling in the United States, 2001–2009: Evidence from the National Household Travel Surveys. *American journal of public health* (2011) 101(S1):S310-S7.

72. Pulakka A, Leskinen T, Suorsa K, Pentti J, Halonen JI, Vahtera J, et al. Physical Activity across Retirement Transition by Occupation and Mode of Commute. *Medicine and science in sports and exercise* (2020) 52(9):1900.

73. Reuvers R, Over EA, Suijkerbuijk AW, Polder JJ, de Wit GA, van Gils PF. Cost-Effectiveness of Mandatory Bicycle Helmet Use to Prevent Traumatic Brain Injuries and Death. *BMC Public Health* (2020) 20(1):1-12.

74. Richter M, Pape H-C, Otte D, Krettek C. The Current Status of Road User Injuries among the Elderly in Germany: A Medical and Technical Accident Analysis. *Journal of Trauma and Acute Care Surgery* (2005) 58(3):591-5.

75. Rodgers GB. Factors Associated with the Crash Risk of Adult Bicyclists. *Journal of Safety Research* (1997) 28(4):233-41.

76. Rolfsman E, Bylund P-O, Saveman B-I. Single Injury Incidents among Pedestrians and Biscyclists in Northern Sweden: Safety and Preventive Issues. *Safety Science Monitor* (2012) 16(1):5.

77. Rosenkvist J, Svensson H, Wretstrand A, editors. How Usable Is the City for Older Bicyclists? *Universal Design 2014, Lund, Sweden, June 16-18, 2014*; 2014: IOS Press.

78. Ryan J, Svensson H, Rosenkvist J, Schmidt SM, Wretstrand A. Cycling and Cycling Cessation in Later Life: Findings from the City of Malmö. *Journal of Transport & Health* (2016) 3(1):38-47.

79. Sadeghvaziri E, Tawfik A, editors. Using the 2017 National Household Travel Survey Data to Explore the Elderly’s Travel Patterns. *International Conference on Transportation and Development 2020*; 2020: American Society of Civil Engineers Reston, VA.

80. Sakurai R, Kawai H, Suzuki H, Ogawa S, Kim H, Watanabe Y, et al. An Epidemiological Study of the Risk Factors of Bicycle-Related Falls among Japanese Older Adults. *Journal of epidemiology* (2019) 29(12):487-90.

81. Sakurai R, Kawai H, Yoshida H, Fukaya T, Suzuki H, Kim H, et al. Can You Ride a Bicycle? The Ability to Ride a Bicycle Prevents Reduced Social Function in Older Adults with Mobility Limitation. *Journal of epidemiology* (2016):JE20150017.

82. Scheiman S, Moghaddas HS, Björnstig U, Bylund P-O, Saveman B-I. Bicycle Injury Events among Older Adults in Northern Sweden: A 10-Year Population Based Study. *Accident Analysis & Prevention* (2010) 42(2):758-63.

83. Shaer A, Haghshenas H. The Impacts of Covid-19 on Older Adults’ Active Transportation Mode Usage in Isfahan, Iran. *Journal of Transport & Health* (2021) 23:101244.

84. Sjögren H, Björnstig U. Injuries to the Elderly in the Traffic Environment. *Accident Analysis & Prevention* (1991) 23(1):77-86.

85. Son SH, Oh SH, Kang SH, Kim D-K, Seo KM, Lee S-U, et al. Independent Factors Associated with Bicycle Helmet Use in a Korean Population: A Cross-Sectional Study. *Traffic injury prevention* (2018) 19(4):399-403.

86. Stones M, Hartin A. Aging and Half-Ironman Performance. *Experimental Aging Research* (2017) 43(2):178-91.

87. Takahashi PY, Baker MA, Cha S, Targonski PV. A Cross-Sectional Survey of the Relationship between Walking, Biking, and the Built Environment for Adults Aged over 70 Years. *Risk Manage Healthc Policy* (2012) 5:35.

88. Tanabe K. Cyclists’ Fractures in the Elderly. *Archives of osteoporosis* (2019) 14(1):1-7.

89. Tredray A. Big Red Ride, Promoting Outdoor Physical Activity and Social Connection for Health-Challenged Older Adults. *Centre for active living* (2019) 30.

90. Tsuji S, Inoue S, Tachibana T, Maruo K, Arizumi F, Yoshiya S. Post-Traumatic Torticollis Due to Odontoid Fracture in a Patient with Diffuse Idiopathic Skeletal Hyperostosis: A Case Report. *Medicine* (2015) 94(36).

91. Tsunoda K, Kitano N, Kai Y, Tsuji T, Soma Y, Jindo T, et al. Transportation Mode Usage and Physical, Mental and Social Functions in Older Japanese Adults. *Journal of Transport & Health* (2015) 2(1):44-9.

92. Tsunoda K, Soma Y, Kitano N, Jindo T, Fujii K, Okura T. Acceptable Walking and Cycling Distances and Their Correlates among Older Japanese Adults. *Journal of Population Ageing* (2021) 14(2):183-200.

93. Tsunoda K, Tsuji T, Kitano N, Mitsuishi Y, Yoon J-Y, Yoon J, et al. Associations of Physical Activity with Neighborhood Environments and Transportation Modes in Older Japanese Adults. *Preventive medicine* (2012) 55(2):113-8.

94. Twisk D, Platteel S, Lovegrove G. An Experiment on Rider Stability While Mounting: Comparing Middle-Aged and Elderly Cyclists on Pedelecs and Conventional Bicycles. *Accident Analysis & Prevention* (2017) 105:109-16.

95. Ulak MB, Ozguven EE, Vanli OA, Dulebenets MA, Spainhour L. Multivariate Random Parameter Tobit Modeling of Crashes Involving Aging Drivers, Passengers, Bicyclists, and Pedestrians: Spatiotemporal Variations. *Accident Analysis & Prevention* (2018) 121:1-13.

96. Useche SA, Alonso F, Montoro L, Esteban C. Distraction of Cyclists: How Does It Influence Their Risky Behaviors and Traffic Crashes? *PeerJ* (2018) 6:e5616.

97. Valent F, Schiava F, Savonitto C, Gallo T, Brusaferro S, Barbone F. Risk Factors for Fatal Road Traffic Accidents in Udine, Italy. *Accident Analysis & Prevention* (2002) 34(1):71-84.

98. Van Cauwenberg J, Clarys P, De Bourdeaudhuij I, Ghekiere A, De Geus B, Owen N, et al. Environmental Influences on Older Adults’ Transportation Cycling Experiences: A Study Using Bike-Along Interviews. *Landsc Urban Plann* (2018) 169:37-46.

99. Van Cauwenberg J, De Bourdeaudhuij I, Clarys P, De Geus B, Deforche B. E-Bikes among Older Adults: Benefits, Disadvantages, Usage and Crash Characteristics. *Transportation* (2019) 46(6):2151-72.

100. Van Cauwenberg J, Clarys P, De Bourdeaudhuij I, Van Holle V, Verté D, De Witte N, et al. Physical Environmental Factors Related to Walking and Cycling in Older Adults: The Belgian Aging Studies. *BMC public health* (2012) 12(1):1-13.

101. Van Cauwenberg J, De Bourdeaudhuij I, Clarys P, De Geus B, Deforche B. Older Adults’ Environmental Preferences for Transportation Cycling. *Journal of transport & health* (2019) 13:185-99.

102. Van Cauwenberg J, De Bourdeaudhuij I, Clarys P, De Geus B, Deforche B. Older E-Bike Users: Demographic, Health, Mobility Characteristics, and Cycling Levels. *Medicine and science in sports and exercise* (2018) 50(9):1780-9.

103. Van Cauwenberg J, Schepers P, Deforche B, De Geus B. Differences in Life Space Area between Older Non-Cyclists, Conventional Cyclists and E-Bikers. *Journal of Transport & Health* (2019) 14:100605.

104. Vance DE, Ross LA, Ball KK, Wadley VG, Rizzo M. Correlates of Individual Physical Activities in Older Adults. *Activities, Adaptation & Aging* (2007) 31(4):1-21.

105. Van den Berg P, Arentze T, Timmermans H. Estimating Social Travel Demand of Senior Citizens in the Netherlands. *Journal of Transport Geography* (2011) 19(2):323-31.

106. Värnild A, Tillgren P, Larm P. Factors Related to the Increasing Number of Seriously Injured Cyclists and Pedestrians in a Swedish Urban Region 2003–17. *Journal of Public Health* (2020) 42(2):e158-e64.

107. Velasco L, Rojo M, Gonzalo-Orden H, Diez JM, editors. Safety Issues with Elderly Cyclists and Barriers to Cycling. *Proceedings of the Institution of Civil Engineers-Municipal Engineer*; 2015: Thomas Telford Ltd.

108. Vlakveld WP, Twisk D, Christoph M, Boele M, Sikkema R, Remy R, et al. Speed Choice and Mental Workload of Elderly Cyclists on E-Bikes in Simple and Complex Traffic Situations: A Field Experiment. *Accident Analysis & Prevention* (2015) 74:97-106.

109. Voss C, Sims-Gould J, Ashe MC, McKay HA, Pugh C, Winters M. Public Transit Use and Physical Activity in Community-Dwelling Older Adults: Combining Gps and Accelerometry to Assess Transportation-Related Physical Activity. *Journal of Transport & Health* (2016) 3(2):191-9.

110. Walsh JM, Pressman AR, Cauley JA, Browner WS. Predictors of Physical Activity in Community-Dwelling Elderly White Women. *Journal of general internal medicine* (2001) 16(11):721-7.

111. Weijermars W, Bos N, Stipdonk HL. Serious Road Injuries in the Netherlands Dissected. *Traffic injury prevention* (2016) 17(1):73-9.

112. Weinz E, Schönle C. Bicycle Accidents in the Aged. *Sportverletzung Sportschaden: Organ der Gesellschaft fur Orthopadisch-traumatologische Sportmedizin* (2001) 15(1):8-11.

113. Weiss R, Juhra C, Wieskötter B, Weiss U, Jung S, Raschke M. Zur Unfallwahrscheinlichkeit Von Senioren Bei Der Nutzung Von E-Bikes. *Zeitschrift für Orthopädie und Unfallchirurgie* (2018) 156(01):78-84.

114. Westerhuis F, Engbers C, Dubbeldam R, Rietman H, Waard DD. Enlightening Cyclists: An Evaluation Study of a Bicycle Light Communication System Aimed to Support Older Cyclists in Traffic Interactions. *International Journal of Human Factors and Ergonomics* (2021) 8(3):294-317.

115. Westerhuis F, Fuermaier AB, Brookhuis KA, de Waard D. Cycling on the Edge: The Effects of Edge Lines, Slanted Kerbstones, Shoulder, and Edge Strips on Cycling Behaviour of Cyclists Older Than 50 Years. *Ergonomics* (2020) 63(6):769-86.

116. Whitaker ED. The Bicycle Makes the Eyes Smile: Exercise, Aging, and Psychophysical Well-Being in Older Italian Cyclists. (2005).

117. Winters M, Voss C, Ashe MC, Gutteridge K, McKay H, Sims-Gould J. Where Do They Go and How Do They Get There? Older Adults' Travel Behaviour in a Highly Walkable Environment. *Social Science & Medicine* (2015) 133:304-12.

118. Winters M, Sims-Gould J, Franke T, McKay H. “I Grew up on a Bike”: Cycling and Older Adults. *Journal of transport & health* (2015) 2(1):58-67.

119. Wong T. Road Traffic Accidents in the Elderly--a Major Cause of Morbidity and Mortality. *Annals of the Academy of Medicine, Singapore* (1987) 16(1):101-5.

120. Yamada Y, Noriyasu R, Yokoyama K, Osaki T, Adachi T, Itoi A, et al. Association between Lifestyle and Physical Activity Level in the Elderly: A Study Using Doubly Labeled Water and Simplified Physical Activity Record. *European journal of applied physiology* (2013) 113(10):2461-71.

121. Zander A, Passmore E, Mason C, Rissel C. Joy, Exercise, Enjoyment, Getting Out: A Qualitative Study of Older People's Experience of Cycling in Sydney, Australia. *J Environ Public Health* (2013) 2013.

122. Zhang Y, Li C, Ding C, Zhao C, Huang J. The Built Environment and the Frequency of Cycling Trips by Urban Elderly: Insights from Zhongshan, China. *J Asian Archit Build Eng* (2016) 15(3):511-8.
